# A modified Ising model of Barabási–Albert network with gene-type spins

**DOI:** 10.1007/s00285-020-01518-6

**Published:** 2020-09-08

**Authors:** Jeyashree Krishnan, Reza Torabi, Andreas Schuppert, Edoardo Di Napoli

**Affiliations:** 1 Aachen Institute for Advanced Study in Computational Engineering Science (AICES) Graduate School, RWTH Aachen University, Aachen, Germany; 2 Joint Research Center for Computational Biomedicine (JRC-Combine), RWTH Aachen University, Aachen, Germany; 3 Department of Physics and Astronomy, University of Calgary, Calgary, AB Canada; 4 Jülich Supercomputing Center, Forschungszentrum Jülich, Jülich, Germany

**Keywords:** Phase transitions, Ising model, Complex networks, Barabási–Albert network, MCMC, Mean-field approximations, 92-10, 82D99, 92B05

## Abstract

The central question of systems biology is to understand how individual components of a biological system such as genes or proteins cooperate in emerging phenotypes resulting in the evolution of diseases. As living cells are open systems in quasi-steady state type equilibrium in continuous exchange with their environment, computational techniques that have been successfully applied in statistical thermodynamics to describe phase transitions may provide new insights to the emerging behavior of biological systems. Here we systematically evaluate the translation of computational techniques from solid-state physics to network models that closely resemble biological networks and develop specific translational rules to tackle problems unique to living systems. We focus on logic models exhibiting only two states in each network node. Motivated by the apparent asymmetry between biological states where an entity exhibits boolean states i.e. is active or inactive, we present an adaptation of symmetric Ising model towards an asymmetric one fitting to living systems here referred to as the modified Ising model with gene-type spins. We analyze phase transitions by Monte Carlo simulations and propose a mean-field solution of a modified Ising model of a network type that closely resembles a real-world network, the Barabási–Albert model of scale-free networks. We show that asymmetric Ising models show similarities to symmetric Ising models with the external field and undergoes a discontinuous phase transition of the first-order and exhibits hysteresis. The simulation setup presented herein can be directly used for any biological network connectivity dataset and is also applicable for other networks that exhibit similar states of activity. The method proposed here is a general statistical method to deal with non-linear large scale models arising in the context of biological systems and is scalable to any network size.

## Introduction

Biological networks are multi-dimensional complex systems whose collective interaction in response to perturbations may lead to critical transitions from one stable state to another. Acute asthma attacks, clinical depression, diabetes mellitus, inflammation, and epileptic seizures, among many others, are examples of such sudden shifts in the state of the system, from healthy to diseased states (Trefois et al. 2015; Wolf et al. 2018). Such “phase transitions” are common in other complex systems such as ecological systems (for example, spontaneous extinction of species in response to gradual changes in external conditions) (Capitan and Cuesta 2010) or the evolution of human language (for example, the formation of Zipfian properties) (DeGiuli 2019; Vera et al. 2020). However, the idea that a system consisting of simple interacting units may exhibit phase transition was initially motivated by a seminal model of magnetic systems called the Ising model.

The Ising model is one of the simplest and most frequently studied models of cooperative phenomena in statistical mechanics (Ising 1925). The classical Ising model is a discrete, pairwise interacting two-state system proposed to explain the structure and properties of ferromagnetic materials and has been solved exactly for one- and two-dimensional lattices (Onsager 1994). In the Ising model of a two-dimensional lattice, each site carries a spin which may be up or down, and neighboring spins prefer to be parallel to each other. The external field prefers to orient the spins in the direction of the field. The spins align in the same direction at low temperature, and the system exhibits spontaneous magnetization. At high temperatures, the spins align randomly, and the system is paramagnetic.

Since then, Ising models have been extended to study phase transitions occurring in more complicated topologies such as random, small-world and scale-free networks (Albert and Barabasi 2002; Bianconi 2002; Barrat and Weigt 2000; Dorogovtsev et al. 2002; Ferreira et al. 2010; Gitterman 2000; Herrero 2002, 2008; Lopes et al. 2004; Pekalski 2001). For example, Ising models of networks can explain how the opinion of the individual is influenced by their contacts on opinions of people on a given subject (Aleksiejuk et al. 2002; Bartolozzi et al. 2006; Castellano et al. 2009; Contucci 2007; Redner 2017). Further real-world applications of Ising models of networks include socioeconomic problems such as racial segregation in the US, group herding, human culture (Stauffer and Hohnisch 2006); phase transitions in neural networks (Aldana and Larralde 2004); communication in the World Wide Web (Kumar et al. 2000); and systems biology (May and Lloyd 2001; Pastor-Satorras et al. 2015; Pastor-Satorras and Vespignani 2001).

In this regard, the analogy between phase transitions occurring in living systems (such as normal to diseased state transition) and physical systems (such as condensation of water) has been well-motivated (Davies et al. 2011; Holstein et al. 2013; Trefois et al. 2015; Smith 2010). The normal state to cancer state transition has been described as a process similar to the first-order irreversible discontinuous phase transition occurring in physical systems (Facciotti 2013; Jin et al. 2017; Liu et al. 2013; Mojtahedi et al. 2016; Torquato 2010). The central idea is that living systems are open systems in quasi-steady state type equilibrium in continuous exchange with their environment wherein cells behave like a network in heat bath under external perturbations (Pastor-Satorras et al. 2015; Scheffer et al. 2012). They survive by exporting entropy to the environment in exchange for structural order. When a control parameter increases entropy, it causes collective flipping of states, which drives the system to an unstable critical state (or diseased state), thereby leading to phase transitions in living systems. In an Ising model, an analog for such a control parameter could be temperature or magnetic field, which, after a particular critical value, may cause the system to undergo a phase transition (discussed in detail in Sect. 2).

An integral part of such living systems is the biological networks that they are composed of—for example, the gene regulatory networks, protein interaction networks, among many others. A gene regulatory network represents a network of genes that can activate or suppress each other’s expression levels owing to the interaction between the genes or due to the influence of agents external to the cell. One of the widely accepted method and a powerful tool for qualitative analysis of dynamics in gene networks is the Boolean dynamic modeling method. In a Boolean model of a network, nodes may be gene or protein and may either take on or off, indicating their expression levels, concentration, or activity. The relationships between the states of nodes are updated by logical functions or truth tables such as AND, OR, or NOT. Though this is a powerful tool, it requires that all possible states of the system i.e. $2^N$ (where $N$ is the network size) be explicitly calculated for the time evolution of the network.

Further, most complex step scales as $O(N^2)$ in the update schemes, and to our knowledge, the model is usually applied to networks whose sizes are of the order of hundred nodes (Campbell and Albert 2014; Wang et al. 2012; Zhang et al. 2008). This is because building such state transition graphs gets computationally expensive as network size increases due to the exponential dependence of the size of state space on network size, thereby making it challenging to analyze large-scale interconnected biological networks such as, for example, the complete human genome. As a consequence of this, when Boolean models are used for constructing signaling pathways on large and dense networks, the number of optimal solutions explodes, which necessitates alternative techniques from statistical physics and graph theory (Alexppoulos et al. 2010; Mitsos et al. 2009).

For large scale simulations, a simpler model that considers gene network as a system in continuous fluctuation that takes into account the current state of the nodes; and does not depend very much on the microscopic details or specific genomic features and is scalable to large sizes would be appropriate. In the method we propose, we overcome the issue with the scale by changing the way we update the state of nodes in response to external perturbations (such as temperature or magnetic field). Firstly, we consider the gene regulatory network as a system that exists in a quasi-steady state in thermal equilibrium with the environment, which aims to conserve energy as a whole whenever it changes its configuration. We establish an initial configuration for all nodes in the network, which may be all zeros or all ones or a combination of zeros and ones based on prior information. Then we perform a random walk over the configuration space. Specifically, this means that we randomly pick a node and calculate the energy cost for the system if this node were to change its state. The walker then hops from the initial configuration to this new configuration only if the transition probability is energetically favorable for the whole system, else the system retains its initial configuration ((Metropolis et al. 1953) summarized in “Appendix”). Such “clever moves” are repeated multiple times to obtain averages to get the behavior of the system to the external perturbation. The behavior of the system is characterized by the mean of the summed states of the system, referred to as magnetization in the context of the classical Ising model.

This method is an abstraction of the gene network and, therefore, only requires the initial configuration of all nodes of the network and network connectivity. It can calculate large scale response features, interpret gene expression of cells in large repositories, and is scalable to network size. This is a qualitative method that can give insights into overall organizing principles in the network and is capable of predicting coherently working clusters in the network. However, it is important to note that, in essence, the model we propose and the Boolean network model are not directly comparable since the former is a thermodynamic model, and the latter is a logical model. They are merely different ways to look at the same system. In the following Sect. 2, we introduce the modified Ising model and motivate the biological analogs of the Ising model by drawing parallels between physical and biological systems.

## Model description and biological motivation

To describe phase transition in a system, we need to take into account interactions between its parts (Goldenfeld 1992; Kardar 2007). Since systems exhibit universal behavior near the critical point (Torabi and Davidsen 2019; Torabi and Rezaei 2016), a variety of statistical mechanical systems can be simulated by Ising-like models provided that the symmetry properties of the system, the pattern of interaction, and the dimensionality of the system is considered (Landau and Lifshitz 1980).

Given that the Ising model is simple and can predict cooperative behavior wherein each element has two states where the energy of each element depends on its state and that of its neighbors, it has found itself wide applications in addressing complexity in biology in the last century. The central aspect of these studies is that usually, the control parameters and physical properties of the Ising model are amenable to a biological interpretation depending on the target biological system being modeled.

In protein science, for example, a relatively popular adaptation of the Ising model, specifically the one-dimensional variant, is the homozipper, which is used as a simplified statistical thermodynamic model for protein folding. The homozipper is a sequence of multiple repeat proteins where each element of a repeat protein is an identical and independently folding unit that interacts with each other in a nearest-neighbor pairwise manner. The sequence can then be pulled apart like a zipper by mechanical force modeled by temperature. The folding is then a process constrained by the number of identical repeats, the energy of the repeated unit, and the interaction energy between the folded units given by the Hamiltonian of the Ising model (Aksel and Barrick 2009; Millership et al. 2016). This proposition of the Ising model to study order-disorder transitions in protein science extended from one-dimensions to higher dimensions for studying helix to coil transitions, beta-hairpin formation, hydrophobicity in protein chains and downhill folding (Garcia-Mira et al. 2002; Kubelka et al. 2004; Kubelka and Kubelka 2014; Lai et al. 2015; Naganathan and Munoz 2014; Munoz et al. 1997; Irback et al. 1996; Irback and Sandelin 2000; Lobanov and Galzitskaya 2011; Zimm and Bragg 1959).

In immunology, an Ising spin-model equivalent of the idiotypic-anti-idiotypic immunological networks has been shown to exhibit self-organization i.e. formation of large homogeneous domains at high temperatures. In such a system, each spin interacts with its mirror-image spin and the neighbors of the image. In the Ising model of such a system, spin up is synonymous to a proliferation of lymphocytes in an ocean of virgin states while spin-down represents a challenge to the immune system. Thus at low temperatures where there are few challenges and low noise, the system exhibits order. While at high-temperature i.e. lots of diseases, a disordered system is formed wherein the net magnetization synonymous to the activity level of lymphocytes is close to zero (Sahimi and Stauffer 1993).

The applications of the Ising model to study genome organization is not new. Ignoring the unique attributes of individual genes, (Baran and Ko 2006) has shown that transcription polarity in a bacterial chromosome i.e. the preference of genes to be coded in the leading strand of replication and their nature to form co-organized clusters can be modeled by a one-dimensional Ising model. Like the magnetic forces that align adjacent spins when the external magnetic field is applied, one could imagine adhesive pseudo-forces such as nearest-neighbor interaction that cause transcription orientation. The chromosome is then simply a series of spin-like indicator variables like that of the $2^N$ configurations that of the one-dimensional Ising lattice. Each gene is oriented negative or positive, depending on the sign of the open reading frame from which it is transcribed. Such models also allow themselves to be analytically tractable for the study of the effects of gene insertion and deletion. A one-dimensional long-range Ising model has been shown to be a rather robust description of long-range correlations in DNA sequences (Colliva et al. 2014).

Ising models have been used to analyze genetic data from affected sib-pair (ASP) where a data point can be represented as $\pm 1$ corresponding to an allele being shared or not by a sib-pair. The nearest neighbor interaction between adjacent dipoles is analogous to the interaction between adjacent genetic markers on a chromosome. The effect of an applied magnetic field i.e. a point field acting on a given particle is analogous to the effect of a disease gene, causing an increase in allele sharing at nearby locations. For example, in the ASP analysis, the coupling constant and magnetic field are interpreted as the strength of genetic linkage between markers and the effect of disease locus in distorting allele sharing in response to random genetic and environmental effects (Majewski et al. 2001).

Furthermore, Ising-and Potts-based models have been proposed for studying phase transitions occurring in more complicated topologies e.g., conformational restructuring affected by temperature. Here a single DNA strand is modeled as a system if interacting bases with short- and long-range interactions. Further, a set of such DNA strands live on a Cayley tree, which is a tree-like graph where each node has an equal number of branches. The edges of this graph may then take multiple spin values say, $\pm 1, 0$ where the former shows the existence of a Holliday junction, and zero means vacant or no edge. The response of the system to temperature is then analyzed based on a Translation Invariant Gibbs Measure (TIGM) (Rozikov 2017, 2018). To our knowledge, these studies do not find any direct mapping between the Ising parameters such as the temperature, magnetic field, or Boltzmann constant to genetic parameters. For an excellent review of similarities between physical and biological systems, we point the interested reader to (Davies et al. 2011).

More examples of applications of Ising models to biological systems include, but is not limited to, a four-dimensional cellular automaton-like Ising model in which cells transition between normal, proliferative, hypoxic and necrotic states has been used to model the tumorigenesis process which involves a transition between these pre-malignant and malignant cell states (Durrett 2013; Torquato 2010); estimating information transfer between spins occurring in human connectome (Marinazzo et al. 2014); the transition of B-DNA to S-DNA (Ahsan et al. 1998); estimation of differentially expressed genes in cancer patients (Xumeng et al. 2011); and approximation of join expression profiles of genes using a small number of observations (Santhanam et al. 2009).

However, to our knowledge, these models do not take into account two aspects of modeling phase transitions in biological networks that we address in this study. Firstly, the discovery of almost scale-free topologies of biological networks in the last couple of decades (Albert and Barabasi 2002). Secondly, the asymmetric i.e. $0,1$ states of activity that has provided a reasonable approximation of the reality of states in single cells (Cesar-Razquin et al. 2018; Wang et al. 2012). In this study, we address these aspects by proposing a modified Ising model for scale-free networks with gene-type spins. The modified Ising model is an arrangement of genes or proteins in a cell where the interaction between the units could be short-range or long-range given by the connectivity matrix of the network. Each unit can interact with their connections in a pairwise manner. These units may activate or suppress each other, given by the binary state variable. A cell can survive in a healthy state if the majority of the units are in an active state, and if it gains energy from the external environment. However, external perturbations may slowly switch the cell to diseased states where the majority of the units may be inactive. Cell survival is, therefore, dependent on conserving energy and changing configurations only if it has a low cost. The Ising analogs for these external variables—energy and entropy are magnetic field and temperature, respectively. Broadly, these control parameters drive the phase transitions process occurring in the living systems.

In this paper, we establish a numerical and theoretical framework on a simulated scale-free network whose nodes exhibit binary states of activity. We show the conditions under which this network of gene-type spins undergoes phase transition due to the influence of temperature and magnetic field. This framework serves as a benchmark for future studies that aim to test dynamics of the Ising model of biological networks with gene-type spins from public databases. We refer to the model that comes out of this as the modified Ising model; a comparison of this model to the classical Ising model (Ising 1925) is summarized in Table 1.

**Table 1:** Comparison of the classical Ising model with the proposed modified Ising model

Attribute	Classical Ising model	Modified Ising model
Topology	Lattice or grid structure	Scale-free network structure
Node	Magnetic spin-type	Gene-type
Spins	Opposing spin states, $-1,1$	Binary spin states, $0,1$
Adjacency matrix	Matrix of nearest–neigbor interactions only with periodic boundary conditions	matrix of short- and long-range interactions
Coupling constant	Indicates the strength of interaction between a pair of spins—if $J> 0$ it is referred to as ferromagnetic and if $J< 0$ it is referred to as anti-ferromagnetic	Indicates the strength of interaction between genes
Temperature	Physical property that changes the ferromagnetic nature of magnets	Biological analog of any control parameter that increase entropy in the system
Magnetic field	Physical property that causes spontaneous magnetization	Biological analog of any control parameter that increases energy in the system
Order parameter	Net magnetic moment which is equal to the mean number of active spins indicating whether the system is ferromagnetic or paramagnetic	Mean number of active genes indicating whether the system is in a state that is analogous to healthy or diseased
Hamiltonian	Total energy of the system	

The classical Ising model—a model of phase transitions occurring in a magnet defined on a lattice with opposing spins. Here we propose a modified Ising model–a model for phase transitions occurring in scale-free networks with binary state variables

Concretely, the Hamiltonian of the Ising model of such a network reads,

1 $$\begin{aligned} H= -\frac{1}{2} \sum _{i,j=1}^{N}J_{ij}s_is_j- h\sum _{i=1}^{N}s_i\quad J_{ij}= JA_{ij} \end{aligned}$$

where $J$ is the coupling constant specifying the strength of interactions; $A_{ij}$ is the adjacency matrix; $h$ indicates the constant external field; $A_{ij}s_is_j$ is the coupling energy arising due to the interaction between nodes and shows the effect of cooperative behavior; $h\sum _{i}s_i$ is the energy arising due to the effect of magnetic field. The Hamiltonian so formed from these two terms is the total energy of the system. If $J > 0$, neighboring spins prefer to take the same values (referred to as ferromagnetic exchange interaction in a classical Ising model); when $J < 0$, neighboring spins prefer to take opposite values (referred to as anti-ferromagnetic exchange interaction in a classical Ising model). Spins, $s_i, s_j$ can take values $\pm 1$ in the classical Ising model; and 0 and 1 in the modified Ising model.

In contrast, in the modified Ising model, the system is a cell containing interacting genes with a scale-free network topology. It is an open system receiving energy from the environment. The first term in the Hamiltonian (Eq. 3), is the two-body interaction term exhibiting pair-wise interaction between genes. Spin values can take 0 or 1 representing the state of gene sitting on a node in the Barabási–Albert network. If the gene is active, it takes value 1, otherwise their contribution in interacting Hamiltonian is zero. Therefore, only if both genes in a pair-wise connection are active, they contribute to this term. Considering that spin values in the biological model are dimensionless Boolean values, this would be the interacting energy between genes at nodes *i* and *j*. The second term in the Hamiltonian is a one-body interaction term exhibiting the interaction of genes with the environment. The critical point in living systems, as open systems, is their interactions with the environment. Therefore, $h$ in the modified Ising model represent this interaction and is the interaction energy between genes and the environment.

The higher $h$ it is, the higher the interaction of the cell with the environment. This parameter (which corresponds to the magnetic field in the ferromagnetic Ising model) tries to retain active genes based on its interactions outside the cell. Minimizing energy in Eq.  3 corresponds to having active genes or healthy state as a result of those mentioned above pair-wise and environmental interactions. The temperature, on the other hand, induces fluctuation that results in randomness in the state of genes. The order parameter is then defined as the number of active genes in the cell,

2 $$\begin{aligned} M= \frac{1}{N} \sum _{i=1}^{n}s_i \end{aligned}$$

In the next sections, we will see how this system experiences a first-order phase transition due to the change in the parameter $h$ whose critical value influence on the properties of the connectivity structure of the genes in the cell. Though the modified Ising model is proposed here with an eye on gene and protein interaction networks, the observations made here, in principle, hold for other similar real-world networks as well. Preliminary results of this work have been presented in the form of a poster and talk (Krishnan et al. 2018, 2019; Krishnan 2019). The paper is organized as follows: Sect. 2 provides a short overview of the Ising model and terminologies used in the subsequent sections of the paper along with biological motivation; in Sect. 3 we show the conditions under which the modified Ising model can undergo phase transitions for different initial configurations of the system (for positive and negative coupling constants) using Monte Carlo simulations; Sect. 4 presents the mean-field solutions and shows a mapping between classical Ising model of scale-free networks and the modified Ising model.

## Numerical simulations

As motivated in Sect. 1 the focus of this paper is to study the system in Eq. 3 for modified Ising spins of the Barabási–Albert network. Such a network is constructed based on two main properties of a real-world network - linear growth and preferential attachment (Albert and Barabasi 2002). The network is initialized with $m_0$ nodes that are not connected. Subsequently, new nodes with $m$ edges are added by an iterative growth process to the existing $m_0$ nodes. The resultant network has a power-law degree distribution and is characterized by a degree exponent, $2< \gamma < 3$ that resembles real-world biological networks.

We now put modified Ising spins on nodes of a Barabási–Albert network of size, $N= 5 \times 10^{3}$ and preferentially attached links, $m= 5$. We choose this network size since it lies in a similar order of magnitude ($\approx 10^3$), such as that of gene regulatory network of standardized datasets such as S. *cerevisiae* or *E*. *coli* (Balaji et al. 2006; Gama-Castro et al. 2008). The number of links attached to grow the Barabási–Albert network is a free parameter and cannot, to our knowledge, be directly compared to real-world networks. Nevertheless we show the effect of the Barabási–Albert model parameters on the modified Ising model in the subsequent sections (cf. Fig. 8).

**Fig. 1:**
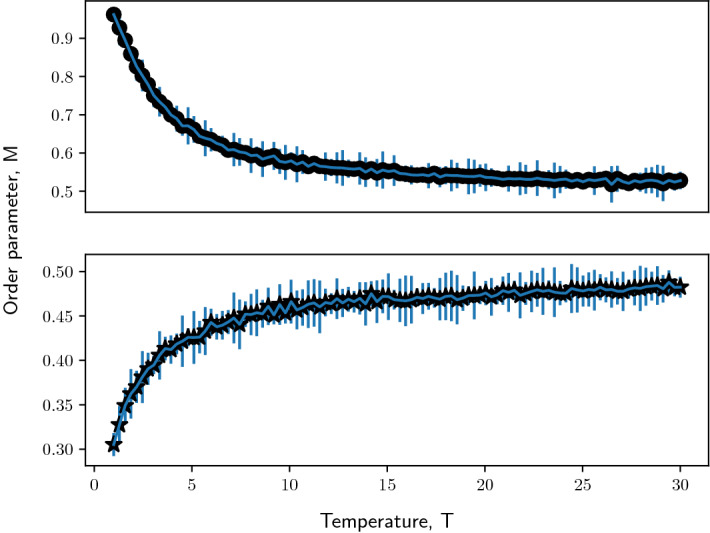
Monte Carlo simulations of the modified Ising model of a Barabási–Albert network at magnetic field, $h= 0$. Figure shows evolution of order parameter, $M$ as a function of Temperature, $T$. Top panel: modified Ising model of Barabási–Albert network with positive coupling constant, $J$ (indicated by black dots). Bottom panel: modified Ising model of Barabási–Albert network with negative coupling constant, $- J$ (indicated by black stars). Simulation parameters: network size, $N= 5 \times 10^3$, preferentially-attached links to construct Barabási–Albert network $m= 5$, magnitude of coupling constant, $|J| = 1$

**Fig. 2:**
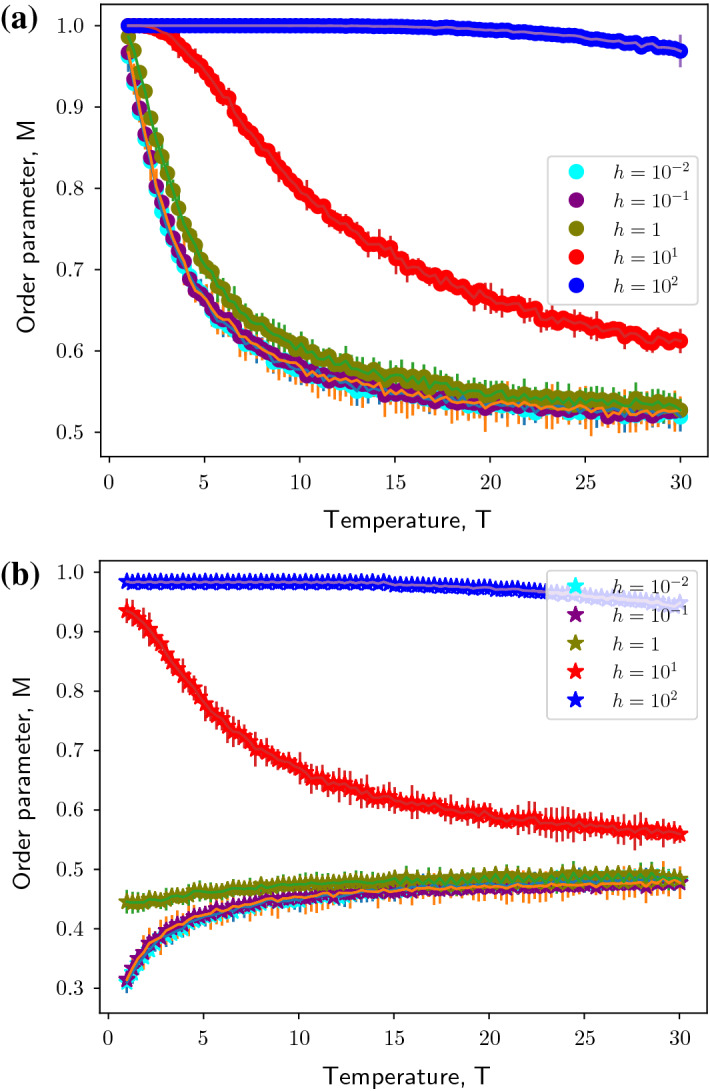
Monte Carlo simulations of the modified Ising model of a Barabási–Albert network in the presence of a positive magnetic field, $h> 0$ of different magnitudes. Figure shows evolution of order parameter, $M$ as a function of Temperature, $T$ for $n = 20$ realizations of the Barabási–Albert network. **(a)** modified Ising model of Barabási–Albert network with positive coupling constant, $J$ (indicated by dots). **(b)** modified Ising model of Barabási–Albert network with negative coupling constant, $- J$ (indicated by stars). Simulation parameters: network size, $N= 5 \times 10^3$, preferentially-attached links to construct Barabási–Albert network, $m= 5$ and magnitude of coupling constant, $|J| = 1$

**Fig. 3:**
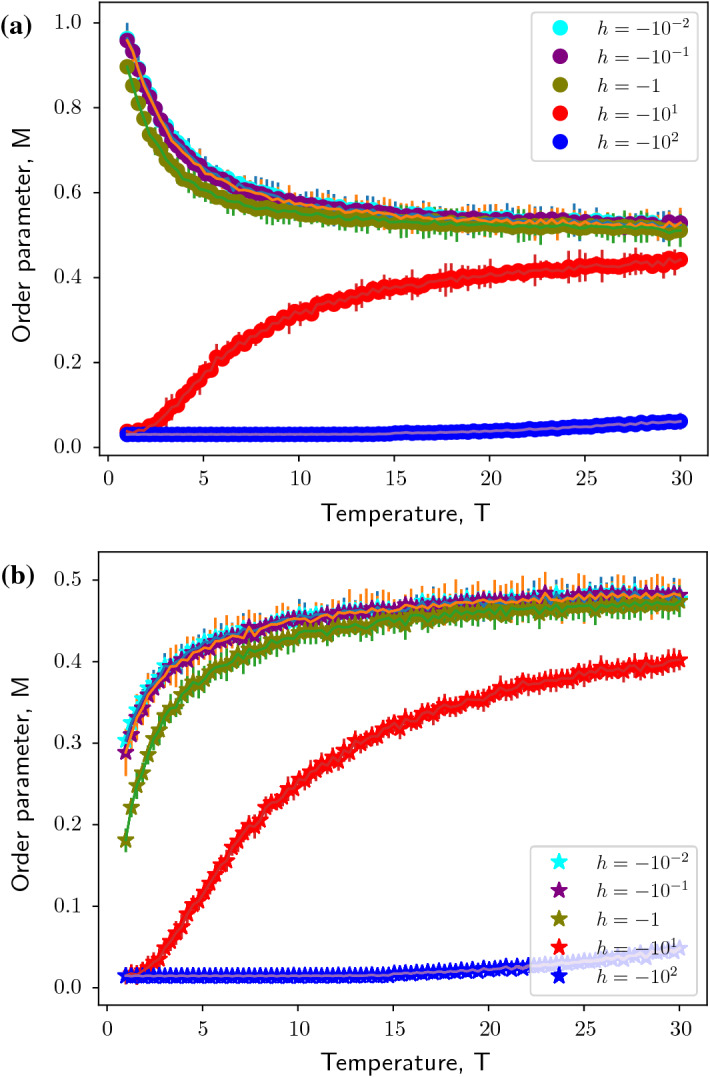
Monte Carlo simulations of the modified Ising model of a Barabási–Albert network in the presence of a negative magnetic field, $h< 0$ of different magnitudes. Figure shows evolution of order parameter, *M* as a function of Temperature, $T$ for $n = 20$ realizations of the Barabási–Albert network. **(a)** modified Ising model of Barabási–Albert network with positive coupling constant, $J$ (indicated by dots). **(b)** modified Ising model of Barabási–Albert network with negative coupling constant, $- J$ (indicated by stars). Simulation parameters: network size, $N= 5 \times 10^3$, preferentially-attached links to construct Barabási–Albert network $m= 5$, magnitude of coupling constant, $|J| = 1$

Then with the standard heat bath Monte Carlo algorithm, we do a spin search for thermal equilibrium at temperature $T$ (cf. algorithm in “Appendix”). The number of equilibration and sampling steps is proportional to the size of the network and has been chosen such that the system has had sufficient time to evolve from its initial configuration and reach a steady state. In other words, the system has visited different states in the phase space and can now generate states that are consistent with the parameters controlling the system. This can be verified by plotting properties of the system, such as magnetization, until it plateaus at a fixed value. We equilibrate the system for $2 \times 10^4$ MC steps and after this temporary period, we simulate for $3 \times 10^4$ MC steps. This allows for an average 10 spin flips per spin. We then sample at the end of every step and perform simulations for both ferromagnetically and anti-ferromagnetically coupled networks, under the influence and absence of the magnetic field.

Under no influence of the magnetic field and ferromagnetic exchange interaction, all nodes in the network start at an active state where the order parameter, $M= 1$. At $T< 1$, the system favors order as seen in the top panel of Fig. 1. As the thermal fluctuations in the system increases, the disorder in the system increases. The order parameter reaches $\frac{1}{2}$ asymptotically as $T \rightarrow \infty$. Similarly, when the system is initialized with an anti-ferromagnetic exchange interaction, the order parameter asymptotically reaches $\frac{1}{2}$ as thermal fluctuations increases (as can be seen in the bottom panel of Fig. 1 at $T<1$).

Under the influence of magnetic field, the system behavior changes as seen in Figs. 2 and 3. Consider the ferromagnetically coupled modified Ising model of Barabási–Albert network influenced by positive magnetic field (Fig. 2a). The field term in the Hamiltonian is effectively a constant holding the network above the mean of two states at $\frac{1}{2}$. As the magnitude of the magnetic field increases, the network takes longer to reach the asymptotic state. For an anti-ferromagnetically coupled modified Ising model of Barabási–Albert network, it can be observed for that for small magnitudes of the positive magnetic field ($h<< 1$), the asymptotic property of order parameter vanishes as in the case of a ferromagnetically-coupled system [Fig. 2b].

**Fig. 4:**
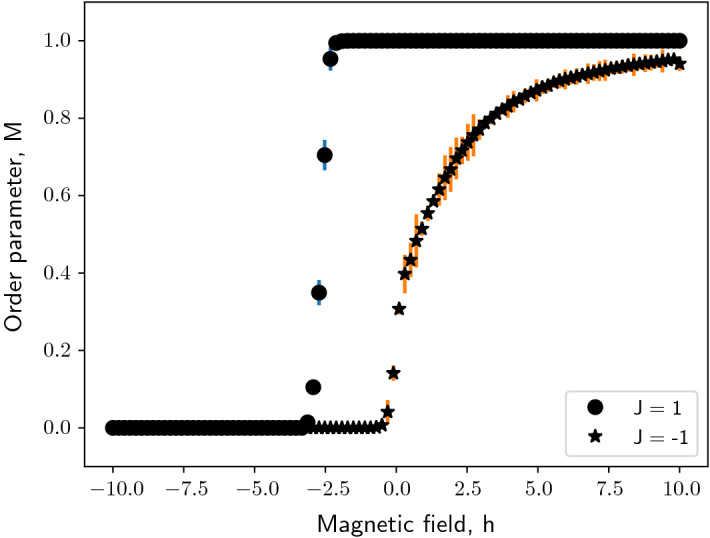
The modified Ising model of a Barabási–Albert network exhibits phase transition under the influence of magnetic field at a fixed Temperature, $T= 0.1$ for $n = 20$ realizations of the Barabási–Albert network. Black dots indicate the order parameter trend for a modified Ising model of Barabási–Albert network with positive coupling constant, $J= 1$. Black stars indicate the order parameter trend for a modified Ising model of Barabási–Albert network with negative coupling constant, $J= -1$

However, for higher magnitudes of the magnetic field, it can be seen that the field term can trigger activity in the network i.e. switch from $M= 0$ to $M= 1$ at $0< T< 1$ and subsequently follow the dynamics of a ferromagnetically-coupled system. A negative magnetic field, on the other hand, inverts the dynamics of a ferromagnetically-coupled modified Ising model instead. As can be seen in Fig. 3a, at $-2.5<h<0$ there is an abrupt drop in the order parameter to 0 and for lower values the network remains inactive (as can be verified from our observations in Figs. 2 and 3). An anti-ferromagnetically coupled network has order parameter $M=0$ at $h=0$. Lower values of the magnetic field keep the network in the inactive state. For a positive magnetic field, the network undergoes a relatively smooth (almost abrupt) phase transition to the active state. Owing to this, unlike in a ferromagnetically coupled network, it can be observed that intermediate values of order parameter and $M\rightarrow 1$ as $h$ increases, confirming our observations in Figs. 2 and 3. Thus it can be inferred that the modified Ising model of a Barabási–Albert network undergoes phase transition due to the magnetic field as shown in Fig. 4.

It can be observed that the transition has a discontinuity in order parameter, and hence this may be a first-order phase transition. Hysteresis loops characterize systems that undergo a first-order phase transition. It implies that the network may show more than one value of order parameter for a given magnetic field, $h$. The hysteresis loop shows the dependence of the state of the system on its history, and it is this phenomenon that forms memory in a hard disk drive.

The procedure to investigate the existence of hysteresis has been well-established, particularly in the context of magnetic materials. We apply the same method for the modified Ising model of a Barabási–Albert network summarized shortly here. Starting with a high negative magnetic field, $h$, and a stable configuration of the system, we increase the magnitude of the magnetic field slowly. For some value of $h$, the local field for a node flips. This causes changes in the effective field of the nodes connected to this node, thereby causing them to flip. Once the flipping in the system has thermalized, the order parameter of the system is measured. Subsequently, the magnetic field is increased slightly, and the process repeated until the order parameter attains a stable state. This way, one can obtain one half of the hysteresis loop (for $h$ from $-\infty$ to $\infty$). The other half of the hysteresis loop is obtained when the magnetic field, $h$ is decreased (for $h$ from $\infty$ to $-\infty$).

**Fig. 5:**
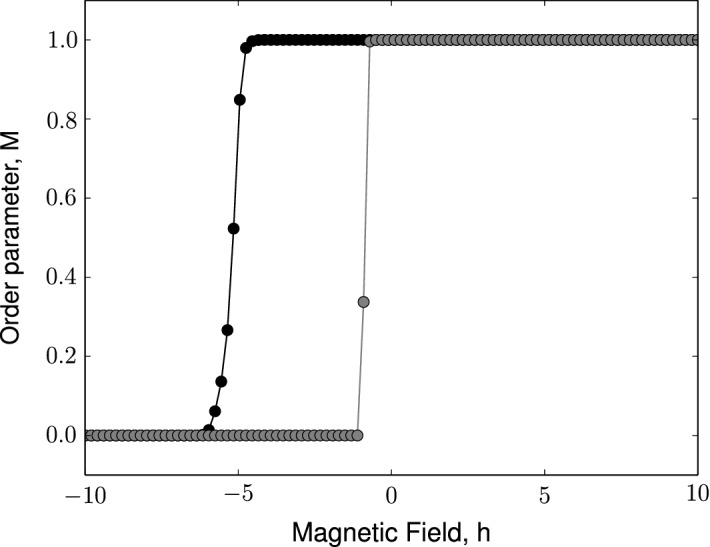
The modified Ising model of a Barabási–Albert network exhibits hysteresis: ferromagnetically coupled, $J= 2$ (indicated by dots). Simulation parameters: $N= 5 \times 10^3$ and preferentially attached links, $m= 5$. The gray curve indicates order parameter as the system is driven forward from $h_0 = 10$ to $h_n = -10$ and the black curve as the system is driven backward from $h_0 = -10$ to $h_n = 10$

A typical hysteresis loop takes the form of a sigmoid; however, in the case of a ferromagnetically coupled modified Ising model  the loop is almost a rectangle, as can be seen in Fig. 5a. We will analyze these observations and discuss the asymptotic behavior in detail using analytical approaches in Sect. 4.

## Analytical methods

### Mean field approximation

One of the most important analytical tool to study disordered systems is represented by mean-field theories. Mean field theory is frequently used due to its conceptual simplicity, as a useful tool, especially when there is no exact solution for the problem. This approximation is used to reduce an interacting problem to a non-interacting one which is easier to solve.

#### Theorem 1

The critical magnetic field of the modified Ising model of a Barabási–Albert network scales linearly with coupling constant and preferentially attached links used to construct the network.

#### Proof

Let us consider the modified Ising model of a Barabási–Albert network treated numerically in Sect. 3. Rewriting the Hamiltonian of the ferromagnetically-coupled system with gene-type spins $0,1$,

3 $$\begin{aligned} H_{0,1} = -\frac{1}{2}\sum _{i,j=1}^{N}J_{ij}s_is_j- h\sum _{i=1}^{N}s_i\quad s_i= 0,1\quad J> 0 \end{aligned}$$

where $J_{ij}= JA_{ij}$. Since the adjacency matrix $A_{ij}$ is symmetric, the factor $\frac{1}{2}$ is included so as not to count any pairs twice. We can write the interactions between neighboring spins in terms of their deviations from the average spin $M$ as,

4 $$\begin{aligned} \begin{aligned} s_is_j&= [(s_i- M) + M][(s_j- M) + M]\\&= (s_i- M)(s_j- M) + M(s_j- M) + M(s_i- M)+ M^2\\ \end{aligned} \end{aligned}$$

where $M= \frac{1}{N}\sum _{i=1}^{N}s_i$ is the order parameter. Assuming that the fluctuations around the mean spin is small, the Hamiltonian can be rewritten as,

5 $$\begin{aligned} \begin{aligned} H_{MF}&= -\frac{1}{2} \sum _{i,j=1}^{N}J_{ij}[M(s_j- M) + M(s_i- M) + M^2] - h\sum _{i=1}^{N}s_i\\&= -\left[ \frac{Jm}{2} \sum _{i=1}^{N} \sum _{j=1}^{N} A_{ij}s_i+ \frac{JM}{2} \sum _{i=1}^{N}\sum _{j=1}^{N}A_{ij}s_j- \frac{JM^2}{2} \sum _{i,j=1}^{N} A_{ij}\right] -h\sum _{i=1}^{N}s_i\\ \end{aligned}\nonumber \\ \end{aligned}$$

Consider the second term in the right hand side of Eq. 5. This can be written as $(i \rightarrow j)$:

6 $$\begin{aligned} \frac{JM}{2} \sum _{i=1}^{N}\sum _{j=1}^{N}A_{ji}s_i= \frac{JM}{2}\sum _{i=1}^{N}\sum _{j=1}^{N}A_{ij}s_j \end{aligned}$$

since $A_{ij}= A_{ji}$, *A* is symmetric. Therefore from Eqs. 5 and 6,

7 $$\begin{aligned} H_{MF}= \frac{JM^2}{2}\sum _{i,j}^{N}A_{ij}- JM\sum _{i,j}^{N} A_{ij}s_i- h\sum _{i=1}^{N}s_i \end{aligned}$$

This is the mean-field Hamiltonian for a chosen realization of the network. So the ensemble average of the Hamiltonian of the system is,

8 $$\begin{aligned} \langle H_{MF}\rangle = \frac{JM^2}{2} \sum _{i,j}^{N} \langle A_{ij}\rangle - JM\sum _{i,j}^{N}\langle A_{ij}\rangle s_i- h\sum _{i=1}^{N}s_i \end{aligned}$$

For a Barabási–Albert network,

9 $$\begin{aligned} \langle A_{ij}\rangle = p_{ij}= \frac{1}{2mN}k_{i}k_{j} \end{aligned}$$

where $k_{i}$ is the number of links of the *i*th node of the network Bianconi (2002) (cf. “Appendix”). From Eqs. 8 and 9, using the relation $\sum _{i=1}^{N}k_{i} = \sum _{j=1}^{N} \approx 2mN$,

10 $$\begin{aligned} \begin{aligned} \langle H_{MF}\rangle&= \frac{JM^2}{2}\sum _{i,j=1}^{N} \frac{1}{2mN}k_{i}k_{j} - Jm\sum _{i,j=1}^{N} \frac{1}{2mN}k_{i}k_{j}s_i- h\sum _{i=1}^{N}s_i\\&= \frac{JM^2}{4mN}\sum _{i=1}^{N}k_{i}\sum _{j=1}^{N}k_{j} - \frac{JM}{2mN}\sum _{j=1}^{N}k_{j}\sum _{i=1}^{N}k_{i}s_i- h\sum _{i=1}^{N}s_i\\&= \frac{JM^2}{4mN} \times 2mN\times 2mN- JM\sum _{i=1}^{N} k_{i}s_i- h\sum _{i=1}^{N}s_i\\&= JM^2 mN- \underbrace{(h+ Jmk_{i})}_{h^{\mathrm {eff}}_{i}}s_i\\ \langle H_{MF}\rangle&= JM^2mN- \sum _{i=1}^{N} h^{\mathrm {eff}}_{i}s_i, \quad h^{\mathrm {eff}}_{i} = (h+ Jmk_{i})\\ \end{aligned} \end{aligned}$$

Hence the modified Ising model of a Barabási–Albert network reduces to a system of non-interacting spins in an effective local field, $h^{\mathrm {eff}}_{i} = (h+ Jmk_{i})$. The partition function can be evaluated as,

11 $$\begin{aligned} Z&= \sum _{\mathrm {config}} e^{-\beta \langle H_{MF}\rangle }\nonumber \\&= \sum _{s_i= 0,1} \ldots \sum _{s_{N} = 0,1} e^{-\beta \Big [ JM^2mN- \sum _{i=1}^{N} h^{\mathrm {eff}}_{i}s_i\Big ]}\nonumber \\&= e^{-\beta JM^2 mN} \prod _{i} \Big ( \sum _{0,1} e^{\beta h^{\mathrm {eff}}_{i}}s_i\Big )\\ Z&= e^{-\beta JM^2 mN} \prod _{i} \Big ( 1 + e^{\beta h^{\mathrm {eff}}_{i}}\Big ) \nonumber \end{aligned}$$

The mean spin, $M$ can be calculated from the partition function using the following relation:

12 $$\begin{aligned} \begin{aligned} M&=\frac{1}{N} \sum _{i=1}^{N}s_i\\&= \frac{1}{N\beta } \frac{\partial \ln Z}{\partial h}\\ \end{aligned} \end{aligned}$$

From this, evaluating $\ln Z$,

13 $$\begin{aligned} \ln Z= - \beta JM^2mN+ \sum _{i} \ln \Big [ 1 + e^{\beta (h+ JMk_{i})}\Big ] \end{aligned}$$

Therefore from Eqs. 12 and 13,

14 $$\begin{aligned} M= \frac{1}{N} \sum _{i=1}^{N}\frac{e^{\beta (h+ JMk_{i})}}{1 + e^{\beta (h+ JMk_{i})}} \end{aligned}$$

$$\square $$

Therefore the central mean-field equation for ferromagnetically coupled Barabási–Albert network with asymmetric spins takes the implicit form,

15 $$\begin{aligned} M= \frac{1}{N} \sum _{i=1}^{N} \frac{1}{1+e^{-\beta (h+ JMk_{i})}} \end{aligned}$$

Similarly for anti-ferromagnetically coupled Barabási–Albert network $(J\rightarrow -J)$ the central mean-field equation is,

16 $$\begin{aligned} M= \frac{1}{N} \sum _{i=1}^{N} \frac{1}{1+e^{-\beta (h- JMk_{i})}} \end{aligned}$$

**Fig. 6:**
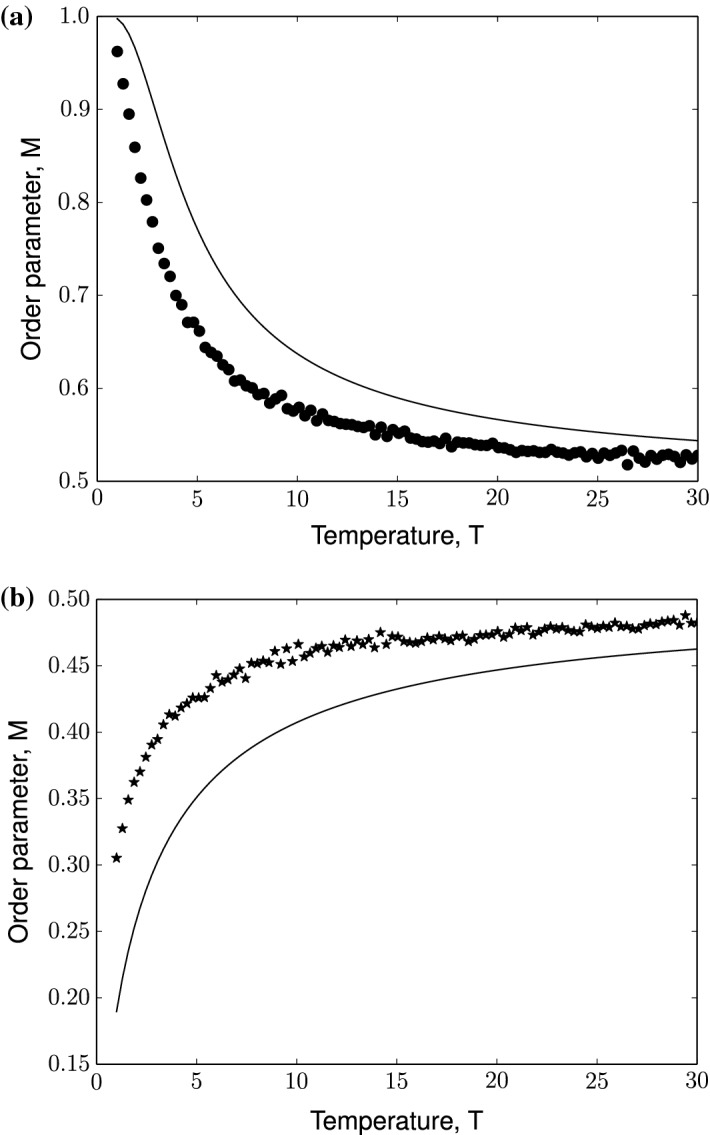
Mean-field theory validates observations from numerical simulations for a modified Ising model of a Barabási–Albert network in the absence of magnetic field. Figure shows evolution of order parameter, $M$ as a function of Temperature, $T$: **(a)** for a modified Ising model of Barabási–Albert network with positive coupling constant, $J$. Black dots indicate Monte Carlo sampling points for $n = 20$ realizations of the Barabási–Albert network. Black curve indicates the trend predicted by the central mean-field equation. **(b)** for a modified Ising model of Barabási–Albert network with negative coupling constant, $-J$. Black stars indicate Monte Carlo sampling points for $n = 20$ realizations of the Barabási–Albert network. Black curve indicates the trend predicted by the central mean-field equation. Simulation parameters: network size, $N= 5 \times 10^3$, preferentially-attached links to construct Barabási–Albert network  $m= 5$, magnitude of coupling constant, $|J| = 1$

Note that the order parameter depends on the coupling constant, $J$ and node degree, $k_{i}$. Let us first study the behavior of the system in the absence of magnetic field. The mean-field equation for ferromagnetically coupled Barabási–Albert network with gene-type spins and no external field is,

17 $$\begin{aligned} M= \frac{1}{N}\sum _{i=1}^{N} \frac{1}{1+e^{\pm \beta JMk_{i}}} \end{aligned}$$

where ± stands for ferromagnetically and anti-ferromagnetically coupling respectively. From Eq. 17 we can investigate the asymptotic behavior for ferromagnetically and anti-ferromagnetically coupled modified Ising model of a network. For a ferromagnetically coupled Barabási–Albert network  when $T\rightarrow \infty$, $\beta JMk_{i} \rightarrow 0$, so $\exp (-\beta JMk_{i}) \rightarrow 1 \implies M\rightarrow \frac{1}{2}$. As $T\rightarrow 0$, $\beta JMk_{i} \rightarrow \infty$, so $\exp (-\beta JMk_{i}) \rightarrow 0 \implies M\rightarrow 1$. These confirm the observations in the top panel in Fig. 1. Similarly for an anti-ferromagnetically coupled modified Ising model of a Barabási–Albert network we can verify the limit cases: as $T\rightarrow \infty , \beta JMk_{i} \rightarrow 0, \exp (\beta JMk_{i}) \rightarrow 1 \implies M\rightarrow \frac{1}{2}$. On the other hand, as $T\rightarrow 0$, $\exp (\beta JMk_{i}) \rightarrow \infty \implies M\rightarrow 0$. These validate the observations in the bottom panel of Fig. 1. In order to compare the results of mean-field approximation with Monte Carlo simulations, we have plotted the results using these two different approaches in Fig. 6 which also shows a slight discrepancy between the numerical simulations and the mean-field ansatz. This arises because we neglect the spin product term in the mean-field approximation with the assumption that the fluctuations around the mean spin is small (Eq. 4).

**Fig. 7:**
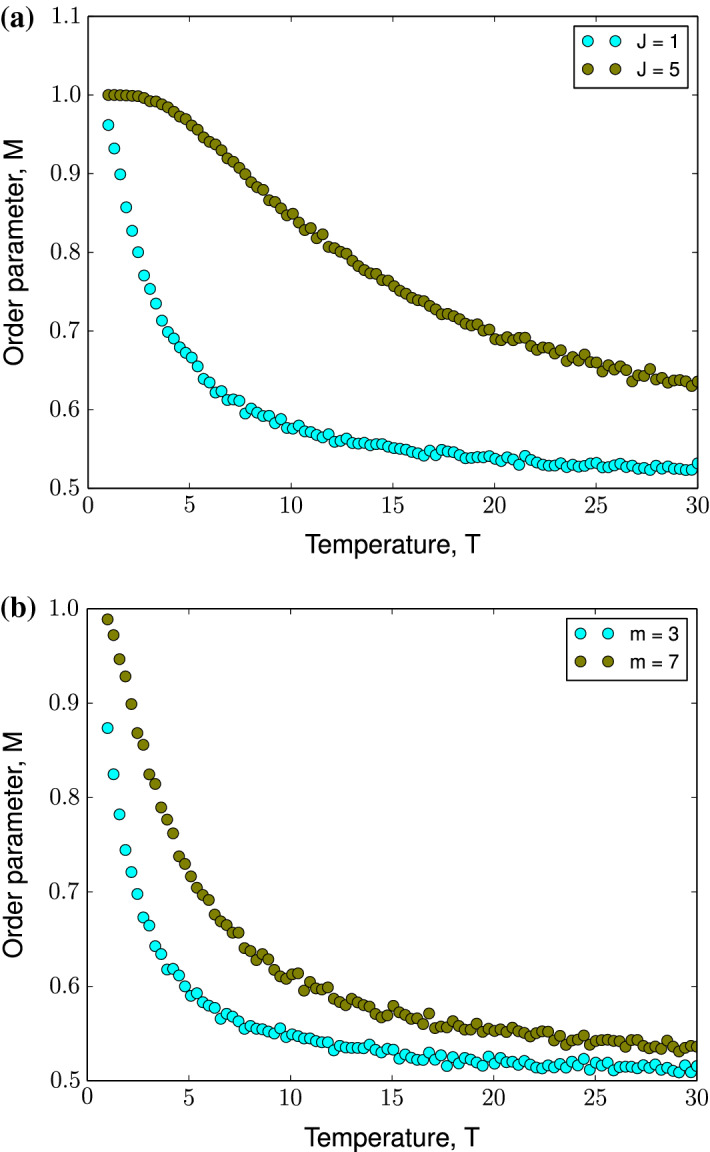
Monte Carlo simulations of modified Ising model of a Barabási–Albert network of size, $N= 5 \times 10^3$ at $h= 0$ and positive coupling constant, $J$ for $n = 20$ realizations of the Barabási–Albert network. **(a)** for coupling constants, $J= 1$ and $J= 5$ with $m= 3$. **(b)** for different choice of preferentially attached links, $m= 3$ and $m= 7$ with $J= 1$. Simulation parameters: network size, $N= 5 \times 10^3$, preferentially-attached links to construct Barabási–Albert network $m= 5$, magnitude of coupling constant, $|J| = 1$

For $T>> 1$, using Taylor expansion $M$ can be approximated as, $M\approx \frac{1}{2\pm \beta Jm}$. We can conclude that, for a fixed large $T$ in ferromagnetically coupled systems, those with larger $J$ and $M$ have larger $M$ and vice versa. This investigation predicts the behavior of the system presented in Fig. 7 and validates Monte Carlo simulations. The situation is reversed for an anti-ferromagnetically coupled system due to the presence of plus sign in the denominator. Eqs. 15 and 16 indicates that at $T>> 1$,

18 $$\begin{aligned} M\approx \frac{1}{2}\Bigg [ \frac{2 + \beta h}{2 \pm \beta Jm}\Bigg ] \end{aligned}$$

However, in both cases, the asymptotic behavior of the system is preserved, for $T\rightarrow \infty$ (or $\beta \rightarrow 0), M\rightarrow \frac{1}{2}$ (cf. Eq. 18). In the case where $T$ not tending to $\infty$, the value of $M$ depends on the magnitude and direction of magnetic field, $h$. This implies that for an anti-ferromagnetically coupled system, when $h> Jm$ then $m> \frac{1}{2}$; however, for $h< Jm$ we have $m> \frac{1}{2}$. Similar conclusions can be made when $h$ is negative in a ferromagnetically coupled system. Therefore, the behavior of the system changes at $|h_c| =Jm$.

Getting back to our biological model, with $J> 0$, when the majority of the genes are active above the critical $h(h_c)$, this represents a healthy state. On the other hand, when the majority of cells are inactive below the critical $h(h_c)$, this represents a disease state. This different behaviour is also illustrated in Fig. 2.

In other words, consider the limit of small $T$ where we can neglect fluctuations. In this case, our biological system, have an order parameter 1 for $h_c> JM$ and 0 for $h_c< JM$. Therefore, above critical parameter $h_c$, all the genes are active while they suddenly become inactive below $h_c$ as a result of a first-order phase transition. This critical external energy interaction depends on the strength of internal interaction (coupling constant $J$ in gene-gene interactions) as well as the parameter $m$ of the Barabási–Albert network.

**Fig. 8:**
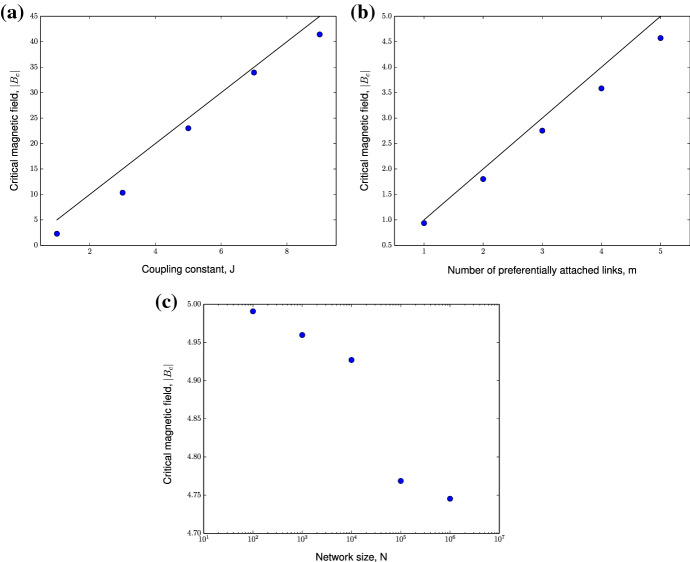
Dependence of critical magnetic field, $h_c$ on network parameters for a modified Ising model of a Barabási–Albert network of size $N= 5 \times 10^3$ with positive coupling constant, $J$ for $n = 20$ realizations of the Barabási–Albert network. **(a)** Coupling constant, $J$ with fixed $m= 5$. **(b)** Number of preferentially attached links to construct Barabási–Albert network, $m$ with fixed $J= 1$. **(c)** on network size, $N$ with other simulation parameters fixed to $J= 1$ and $m= 5$. Blue dots indicate results from Monte Carlo simulations and black line indicates analytical results

This approximates the critical magnetic field, $h_c\approx 5$ for the choice of simulation parameters, which is very close to our observations from numerical simulations as can be verified in Figs. 2 and 3. Although the analytical results predict that the network size does not influence phase transition in the modified Ising model of the Barabási–Albert network, the numerical results predict a weak dependence of $h_c$ on network size (Fig. 8c), which appears in systems with large network sizes. The dependence on parameters $J$ and $m$ is over-estimated by the mean-field calculations as can be seen in Fig. 7. In the next Sect. 4.1 we will derive the expression for the critical magnetic field by mapping the modified Ising spin system to the classical spin system on a Barabási–Albert network.

#### Theorem 2

There exists a transformation between a modified Ising model and the classical Ising model.

#### Proof

The numerical and analytical observations presented in Sects. 3 and 4 can be validated by mapping the Hamiltonian of the modified Ising model of Barabási–Albert network  $H_{0,1}$ to the well-established classical Ising spin system on Barabási–Albert network  $H_{-1,1}$. Rewriting the modified Ising model Eq. 3,

19 $$\begin{aligned} H_{0,1} = - \frac{1}{2}\sum _{i,j}^{N}J_{ij}s_is_j- h\sum _{i=1}^{N}s_i\quad s_i= 0,1 \end{aligned}$$

This can be mapped to the Hamiltonian of the classical spin system by introducing new spin variables as,

20 $$\begin{aligned} s_{i}^{\prime }= 2 \Bigg (s_i- \frac{1}{2}\Bigg ) \end{aligned}$$

For $s_i= 0 \rightarrow s_{i}^{\prime }= -1$ and for $s_i= 1 \rightarrow s_{i}^{\prime }= 1$. Substituting the spin variables in the Hamiltonian Eq. 19 we make the $H_{0,1} \rightarrow H_{-1,1}$ transformation,

21 $$\begin{aligned} \begin{aligned} H_{0,1}&= \frac{-1}{2}\sum _{i,j}^{N}J_{ij}s_is_j-h\sum _{i=1}^{N}s_i\quad s_i=0,1\\ H_{-1,1}&= \frac{-1}{2}J_{ij}\Big ( \frac{s_{i}^{\prime }+ 1}{2} \Big ) \Big ( \frac{s_{j}^{\prime }+ 1}{2} \Big ) - h\sum _{i=1}^{N} \Big ( \frac{s_{i}^{\prime }+ 1}{2} \Big ) \quad s^{\prime } = -1,1\\&= \frac{-1}{2} \sum _{i,j}^{N} \frac{J_{ij}}{4}s_{i}^{\prime }s_{j}^{\prime }- \frac{1}{2}\sum _{i,j}^{N} \frac{J_{ij}}{4} (s_{i}^{\prime }+ s_{j}^{\prime }) -\frac{1}{2}\sum _{i,j}^{N}\frac{J_{ij}}{4}-\frac{h}{2}\sum _{i=1}^{N}s_{i}^{\prime }- \frac{h}{2}\\ \end{aligned}\nonumber \\ \end{aligned}$$

Since $J_{ij}= J_{ji}$, $\sum _{i,j}^{N}(s_{i}^{\prime }+ s_{j}^{\prime }) = 2\sum _{i,j}^{N}s_{i}^{\prime }$, eq. 21 can be re-written as,

22 $$\begin{aligned} \begin{aligned} H_{-1,1}&= -\frac{1}{2}\sum _{i,j}^{N}\frac{J_{ij}}{4}s_{i}^{\prime }s_{j}^{\prime }- \sum _{i,j}^{N} \frac{J_{ij}}{2}s_i- \sum _{i,j}^{N}\frac{J_{ij}}{8}-\frac{h}{2}\sum _{i=1}^{N}s_{i}^{\prime }- \frac{h}{2}\\&= -\frac{1}{2}\sum _{i,j}^{N} \underbrace{\frac{J_{ij}}{4}}_{\mathrm {new\ coupling, J_{ij}^{\prime }}}s_{i}^{\prime }s_{j}^{\prime }- \sum _{i=1}^{N} \underbrace{\Big [ \frac{h}{2} + \sum _{j=1}^{N} \frac{J_{ij}}{2}\Big ]}_{\mathrm {new\ local\ magnetic\ field,\ h^{\prime }}}s_{i}^{\prime }- \underbrace{\Big [ \sum _{i,j}^{N} \frac{J_{ij}}{8} + \frac{h}{2}\Big ]}_{\mathrm {constant,} E_0}\\ \end{aligned}\nonumber \\ \end{aligned}$$

So the problem of an Ising model with gene-type spin system is mapped on to a problem of Ising model with classical spin system as,

23 $$\begin{aligned} H_{-1,1} = E_0 - \frac{1}{2} \sum _{i,j}^{N}J_{ij}^{\prime }s_{i}^{\prime }s_{j}^{\prime }- \sum _{i=1}^{N}h^{\prime }_{i}s_{i}^{\prime } \end{aligned}$$

where constant $E_0 = -\sum _{i,j}^{N} \frac{J_{ij}}{8}- \frac{h}{2}$, new coupling $J_{ij}^{\prime } = \frac{J_{ij}}{4}$ and new local magnetic field, $h^{\prime }= \frac{h}{2} + \sum _{j=1}^{N} \frac{J_{ij}}{2}$. The fact that even in the absence of magnetic field there is an intrinsic local magnetic field, a $\sum _{i,j}^{N} \frac{J_{ij}}{2}$ in the system reflects the asymmetricity of the spins present in the problem. In principle, any physical quantity of the system of modified Ising model of a interaction can therefore be derived from the system of Ising spins,

24 $$\begin{aligned} Z_{0,1}(J_{ij}, h) = e^{\beta E_0}Z_{-1,1}(J_{ij}^{\prime }, h^{\prime }_{i}) \end{aligned}$$

However we are interested in the critical magnetic field as derived in Sect. 4.1. Note that, the first term of the right hand of Eq. 23 is a constant and by redefinition of the zero of energy we have,

25 $$\begin{aligned} H= -\frac{1}{2}\sum _{i,j}^{N}J_{ij}^{\prime }s_{i}^{\prime }s_{j}^{\prime }- \sum _{i=1}^{N}h^{\prime }_{i}s_{i}^{\prime } \end{aligned}$$

This is the Hamiltonian for a chosen realization of the network. So the ensemble average of the system Hamiltonian is,

26 $$\begin{aligned} \langle H_{MF}\rangle = -\frac{1}{2}\sum _{i,j}^{N}\langle J_{ij}^{\prime } \rangle s_{i}^{\prime }s_{j}^{\prime }- \sum _{i=1}^{N}\langle h^{\prime }_{i}\rangle s_{i}^{\prime } \end{aligned}$$

where,

27 $$\begin{aligned} \begin{aligned} \langle h^{\prime }_{i} \rangle&= \frac{h}{2} + \frac{-J}{2}\sum _{j=1}^{N}A_{ij}\\&= \frac{h}{2} - \frac{J}{2}\sum _{j=1}^{N}\frac{k_i k_j}{2mN}\\ \langle h^{\prime }_{i} \rangle&= \frac{h}{2}-\frac{J}{2}k_i\\ \end{aligned} \end{aligned}$$

$$\square $$

#### Remark 1

From the above transformation to the classical Ising model, the average critical field for the modified Ising model can be verified.

The average critical field for the system $h_c$ can be derived by,

28 $$\begin{aligned} \frac{h_c}{2}-\frac{J}{2}\bar{k}= 0 \end{aligned}$$

where $k_i$ is approximated by the average number of links, $\bar{k}$. Note that $\bar{k}= \frac{1}{N}\sum _{i=1}^{N}k_i \approx \frac{1}{N}\times 2mN= 2m$, thus,

29 $$\begin{aligned} h_c\approx Jm \end{aligned}$$

This validates our results presented in Sect. 4.1. Eq. 29 predicts that the critical magnetic field depends linearly on $J$ and $m$. The numerical simulations confirms the analytical predictions on critical magnetic field (Fig. 8a and b).

## Conclusions

In living systems, collective flipping of coherently expressed genes is associated with disease progression. This flipping causes the step by a step-change in the phenotype of the cell, causing it to transition from normal phase to diseased phase. Similarly, in magnetic systems, collective flipping of spins is associated with the loss of spontaneous magnetization. Therefore it is intuitive to consider gene networks as two-state thermodynamic systems in a heat bath obeying Boltzmann statistics.

On the one hand, in the statistical physics community, there have been extensive studies on phase transitions occurring in Ising models of scale-free networks with classical spins (discussed in Sect. 1). These methods are analytically tractable and applicable to very large sizes. On the other hand, in the systems biology community, a wealth of literature exists that motivates the modeling of networks with binary states for small to medium sizes (discussed in Sect. 2). This work on modified Ising model lies at the intersection of statistical physics and network biology and is presented with an intention to bring the two schools of thought together.

In this regard, we have proposed here an adaptation of a well-established model in statistical mechanics that could be used to study phase transitions in living systems. This model allows simplification of interactions in complex systems; can be studied analytically; and renders itself adaptable to the representation of complex genetic systems, thereby allowing testing of the diverse hypothesis that may cause complex disorders. The modified Ising model is an adaptation of the classical Ising model constructed for networks with a scale-free-like structure and whose activity is described by a binary random variable. This is a general statistical method to deal with poorly understood non-linear large scale models arising in the context of biological networks.

We have presented a basic numerical and theoretical framework to investigate phase transitions using modified Ising model where the control parameters energy and entropy are modeled by the magnetic field and temperature, respectively. Taking the Barabási–Albert model as the toy model, we have shown that such a system undergoes phase transition owing to the influence of the critical magnetic field. This is synonymous to a simple Mendelian disease where there is a strong field near the diseased gene. In complex diseases, the influence of the magnetic field is spread among different genes with different strengths.

The critical magnetic field of the system scales linearly as a function of the number of preferentially attached links and coupling constant. Further, we have shown that the modified Ising model can be mapped to a classical Ising model of a Barabási–Albert network. The simulation setup presented herein can be directly used for any biological network connectivity dataset and is also applicable to other networks that exhibit similar states of activity. The model can be adapted for directed or weighted networks and could also take a continuum of activity states such as in a Potts model. Additional interaction terms may be added to the Hamiltonian to model epistatic interactions between genes. Further, the modified Ising model is capable of predicting the existence of structurally or functionally organized clusters in the network.

We have shown that a purely qualitative model such as the modified Ising model is capable of predicting phase transitions given only the connectivity without logical rules or kinetic parameter data. This indicates that dynamics on these networks may depend more on structure than on the specific details of the processes. The modified Ising model is capable of scaling to networks of sizes up to tens of thousands and can potentially predict similarities between apparently unrelated complex systems.

## Appendix

### A. Metropolis algorithm

- A random node in the network is picked.
- A trial flip of its spin, is performed and energy cost to the system to change its configuration is then calculated.
- If energy difference is less than zero, it leads to a lower energy state, and hence the network is updated to the new configuration.
- Else, if energy difference is greater than zero, a random number is generated.
- The system is allowed to move to a higher energy state only if the random number is greater than the energy difference.

### B. Approximation of ensemble average of adjacency matrix by network parameters

Here we summarize the approach from Bianconi (2002) to reduce mean adjacency matrix over many realization of Barabási–Albert network to network parameters. Let us consider a Barabási–Albert network of $N$ nodes. Starting from a small number of nodes $n_{0}$ and links $m_{0}$ (where $n_{0}, m_{0}<< N$), the network is constructed iteratively by the constant addition of nodes with $m$ links. The new links are preferentially attached to well connected nodes in such a way that at time $t_j$, the probability $p_{ij}$ that the new node *j* is linked to node *i* with connectivity $k_i(t_j)$ is given by,

30 $$\begin{aligned} p_{ij}= m\frac{k_{i}(t_j)}{\sum _{\alpha = 1}^{j} k_{\alpha }} \end{aligned}$$

is proportional to the number of links $k_i$ at time $t_j$, and number of preferentially attached links $m$. The dynamic solution of connectivity at time $t_i$ is,

31 $$\begin{aligned} k_{i} = m\sqrt{\frac{t}{t_i}} \end{aligned}$$

From Eqs. 30 and 31 we have,

32 $$\begin{aligned} p_{ij}= m\frac{m\sqrt{\frac{t}{t_i}}}{\sum _{\alpha = 1}^{j} k_{\alpha }(t)} \end{aligned}$$

If $N$ is large we can approximate the total number of edges in the network at time $t_j$, given by the sum $\sum _{\alpha =1}^{j}k_{\alpha }$ as,

33 $$\begin{aligned} \sum _{\alpha = 1}^{j} k_{\alpha } = m_0 + 2mt_j \approx 2mt_j \end{aligned}$$

because $m_{0}<< N$. The factor 2 comes from the fact that as we create a link which connects two nodes, the number of links of each of them increases by 1. Substituting Eq. 33 in 32,

34 $$\begin{aligned} \begin{aligned} p_{ij}&= \frac{m^2 \sqrt{\frac{t_j}{t_1}}}{2mt_j}\\&= \frac{m}{2} \frac{1}{\sqrt{t_it_j}}\\ \end{aligned} \end{aligned}$$

The adjacency elements of the network $A_{ij}$ are equal to 1 if there is a link between node *i* and *j* and 0 otherwise. Consequently the mean over many copies of a Barabási–Albert network 

35 $$\begin{aligned} \langle A_{ij}\rangle = p_{ij}= \frac{m}{2}\frac{1}{\sqrt{t_it_j}} \end{aligned}$$

From Eq. 31 we can re-write for $t = N$ steps,

36 $$\begin{aligned} \begin{aligned} k_{i}{(t)}&= m\sqrt{\frac{t}{t_i}}\\ k_{i}(N)&= m\sqrt{\frac{N}{t_i}}\\ t_i&= \frac{m^2 N}{k_{i}^{2}} \end{aligned} \end{aligned}$$

and similarly,

37 $$\begin{aligned} t_j = \frac{m^2N}{k_{j}^{2}} \end{aligned}$$

From Eqs. 36 and 37,

38 $$\begin{aligned} \begin{aligned} \langle A_{ij}\rangle&= \frac{m}{2} \frac{1}{\sqrt{\frac{m^2N}{k_{i}^{2}}}\sqrt{\frac{m^2N}{k_{j}^{2}}}}\\&= \frac{1}{2mN}k_{i}k_{j}\\ \end{aligned} \end{aligned}$$

The average of the adjacency matrix over many realizations can be approximated by the network parameters as,

39 $$\begin{aligned} \langle A_{ij}\rangle = \frac{1}{2mN}k_{i}k_{j} \end{aligned}$$

## List of symbols

$$A_{ij}$$: Adjacency matrix
$h_c$: Critical magnetic field
$\beta$: Inverse of temperature
$$J$$: Coupling constant
$\gamma$: Scale-free exponent
$0,1$: Modified Ising spins
$h^{\mathrm {eff}}$: Effective magnetic field
$$h$$: Magnetic field
$$H$$: Hamiltonian
$H_{MF}$: Mean-field hamiltonian
$$k_B$$: Boltzmann constant
$$k$$: Node degree
$$M$$: Order parameter
$$\bar{k}$$: Mean degree
$m$: Number of preferentially attached links
$$-1,1$$: Classical spins
$$N$$: Network size
$p_{ij}$: Probability that new node is linked to existing node
$$T$$: Temperature
$$Z$$: Partition function
